# Targeting a Reticulocyte Binding Protein and Duffy Binding Protein to Inhibit Reticulocyte Invasion by *Plasmodium vivax*

**DOI:** 10.1038/s41598-018-28757-4

**Published:** 2018-07-12

**Authors:** Sonal Gupta, Shailja Singh, Jean Popovici, Camille Roesch, Ahmed Rushdi Shakri, Micheline Guillotte-Blisnick, Christèle Huon, Didier Menard, Chetan E. Chitnis

**Affiliations:** 1 International Centre for Genetic Engineering and Biotechnology (ICGEB), New Delhi, India; 2 Special Centre for Molecular Medicine, Jawaharlal Nehru University, New Delhi, India; 3 Shiv Nadar University, Gautam Buddha Nagar, India; 4 Institut Pasteur, Paris, France; 5 Institut Pasteur of Cambodia, Phnom Penh, Cambodia

## Abstract

*Plasmodium vivax* merozoite invasion is restricted to Duffy positive reticulocytes. Merozoite interaction with the Duffy antigen is mediated by the *P. vivax* Duffy binding protein (PvDBP). The receptor-binding domain of PvDBP maps to an N-terminal cysteine-rich region referred to as region II (PvDBPII). In addition, a family of *P. vivax* reticulocyte binding proteins (PvRBPs) mediates interactions with reticulocyte receptors. The receptor binding domain of *P. vivax* reticulocyte binding protein 1a (PvRBP1a) maps to a 30 kD region (PvRBP1a_30_). Antibodies raised against recombinant PvRBP1a_30_ and PvDBPII recognize the native *P. vivax* antigens and inhibit their binding to host receptors. Rabbit IgG purified from sera raised against PvRBP1a_30_ and PvDBPII were tested individually and in combination for inhibition of reticulocyte invasion by *P. vivax* field isolates. While anti-PvDBPII rabbit IgG inhibits invasion, anti-PvRBP1a_30_ rabbit IgG does not show significant invasion inhibitory activity. Combining antibodies against PvDBPII and PvRBP1a_30_ also does not increase invasion inhibitory activity. These studies suggest that although PvRBP1a mediates reticulocyte invasion by *P. vivax* merozoites, it may not be useful to include PvRBP1a_30_ in a blood stage vaccine for *P. vivax* malaria. In contrast, these studies validate PvDBPII as a promising blood stage vaccine candidate for *P. vivax* malaria.

## Introduction

Malaria remains a major public health problem in the tropical world^1–3^. While *P. falciparum* infections account for more than 90% of malaria cases in sub-Saharan Africa, *P. vivax* is responsible for majority of malaria cases in many parts of the world outside Africa such as India, South-East Asia, Western Pacific and Latin America^1–3^. Intensified malaria control efforts over the past decade have greatly reduced the number of malaria cases and deaths attributed to malaria. However, in areas of high transmission, gains in reduction of malaria cases have stagnated in recent years with the number of malaria cases remaining around 215 million per year resulting in approximately 450,000 deaths per year in 2015 and 2016^1,2^. It appears unlikely that malaria elimination can be achieved in areas of high transmission with currently available tools. The unique biology of *P. vivax*, which involves the latent hypnozoite stage in the liver, which can activate to yield blood stage infections and cause clinical malaria months or even years after the initial infection, and the early appearance of gametocytes, which enables transmission even before clinical symptoms develop, makes elimination of *P. vivax* malaria more challenging than elimination of *P. falciparum*^4^. The availability of an effective vaccine that protects against vivax malaria and inhibits *P. vivax* transmission can greatly help efforts to control and eliminate malaria^5^.

All the clinical symptoms of malaria are attributed to the blood stages of the infection during which plasmodium merozoites invade and multiply within host red blood cells (RBCs). RBC invasion requires specific molecular interactions between parasite protein ligands on the invading merozoite and receptors on host RBCs^6^. Understanding the key receptor-ligand interactions that mediate host cell invasion can enable the development of a recombinant subunit based malaria vaccine. Such a vaccine would elicit antibodies against key parasite protein ligands to inhibit their interaction with host receptors and block RBC invasion. Vaccines that inhibits blood stage parasite growth with high efficiency may also reduce gametocyte densities and have an impact on malaria transmission. Such a vaccine would not only protect against *P. vivax* malaria but would also interrupt malaria transmission in line with the goal of achieving *P. vivax*elimination^7^.

*P. vivax* blood stage infection is restricted to human reticulocytes compared to *P. falciparum*, which infects mature RBCs^8^. Moreover, *P. vivax* merozoites are primarily dependent on interaction with the Duffy blood group antigen receptor for chemokines (DARC) for reticulocyte invasion^9,10^. *P. vivax* infection in Duffy negative individuals has been recently reported although this is not yet common across sub-Saharan Africa where more than 90% of the population is Duffy negative^11,12^. The 140 kD *P. Vivax* Duffy binding protein (PvDBP) binds DARC to mediate invasion^13^. Given that DARC is expressed both on reticulocytes and mature erythrocytes, the PvDBP-DARC interaction cannot be responsible for the preferential invasion of reticulocytes by *P. vivax*. Specificity for invasion of reticulocytes is attributed to a family of *P. vivax* reticulocyte binding proteins (PvRBPs), which bind receptors on reticulocytes that are lost during erythrocyte maturation^14^. PvRBPs are divided into sub-families referred to as PvRBP1 (composed of PvRBP1a and PvRBP1b) and PvRBP2 (composed of PvRBP2a, PvRBP2b, PvRBP2c) based on sequence homology^15,16^. PvRBP2d and PvRBP3 are pseudo-genes that share homology with PvRBPs but do not encode functional proteins.

PvRBPs share homology with the PfRH family of *P. falciparum* proteins, which bind receptors on erythrocytes to mediate invasion by *P. falciparum* merozoites^15,16^. The receptor binding domains of some of the PfRH and PvRBP proteins have been mapped^16^. Here, we produce the reticulocyte-binding domain of PvRBP1a, which maps to a 30kD region (PvRBP1a_30_) that shares homology with the binding domain of the *P. falciparum* protein PfRH4. We confirm that recombinant PvRBP1a_30_ preferentially binds reticulocytes and test the ability of antibodies raised against recombinant PvRBP1a_30_ to recognize native PvRBP1aantigen as well as block reticulocyte binding by PvRBP1a and reticulocyte invasion by *P. vivax* isolates. In addition, given the key role played by PvDBP in mediating interaction with DARC during invasion we also tested the ability of antibodies raised against PvDBPII to block reticulocyte invasion both individually and in combination with antibodies against PvRB1a_30_. Data from these functional studies will be used to validate individual *P. vivax* antigens as well as antigen combinations for inclusion in a multi-antigen blood stage vaccine candidates for *P. vivax* malaria.

## Materials and Methods

### Mammalian cell culture and transfection

COS7 cells were cultured in Dulbecco Modified Eagle Medium (DMEM) with 10% heat inactivated fetal bovine serum (FBS) (Gibco, Thermofisher Scientific Inc., USA) in a humidified CO_2_ incubator at 37 °C. The cells were seeded in six well plates and transfected at 50–60% confluency with 2 μg of plasmid DNA using lipofectamine. After 24 h of transfection, the cells were used for immunofluorescence and rosetting assays as described earlier^17^.

### Reticulocyte enrichment from human blood and analysis by flow cytometry

Reticulocytes comprise 0.5–1% of cells in human blood. Reticulocytes were enriched from normal human blood by immuno-magnetic separation using magnetic beads (MACS, Miltenyi Biotec) conjugated with anti-CD71 antibodies. Briefly, 10^7^ cells were resuspended in 80 μl MACS buffer (PBS pH 7.2, 0.5%BSA) with 20 μl of anti-CD71 micro-beads (Miltenyi Biotec) and incubated for 15 min at 4 °C. The unbound beads were removed with MACS buffer by centrifuging at 300 × g for 10 min. The anti-CD71 microbeads with bound reticulocytes were applied to a pre-equilibrated magnetic column placed in a magnetic field. Bound reticulocytes were eluted from the column by removing the column from the magnetic field. These enriched reticulocytes were stored in RPMI 1640 at 50% hematocrit at 4 °C. The enrichment of reticulocyte population was estimated by scoring cells stained with Reticulocyte Stain (Sigma Aldrich Pvt. Ltd., USA) by flow cytometry (Supplementary Figure S1). The enriched reticulocytes were used for rosetting assays with transfected COS 7 cells and for binding assays with recombinant proteins.

The purity of reticulocytes was assessed by flow cytometry (BD FACS Calibur) using antibodies against reticulocyte surface marker CD71 conjugated to phycoerythrin (PE). Briefly, 10^7^ reticulocyte enriched erythrocytes were incubated with anti CD71-PE antibodies (1:10 dilution) for 15 mins at 4 °C. Following incubation, the cells were washed and resuspended in 200 µl of PBS and analyzed on FACS using BD CELLQUEST software.

### Expression of PvRBP1a_30_ on the surface of COS 7 cells and use in binding assays

The gene segment of *pvrbp1* encoding the ~30 kD reticulocyte binding domain of PvRBP1a (PvRBP1a_30_, 352–599 aa), was amplified by PCR with genomic DNA of *P. vivax* Belem strain as template using gene specific primers (FP 5′-GACCAGCTGAACGAACTAGGTATAGACATTC-3′ and RP 5′-AACGGGCCCTTCTTTGTAAAATTTTTCCACTG-3′). The PCR product was digested with *Pvu*II and *Apa*I and cloned in the plasmid pRE4 to create a fusion of PvRBP1a_30_ with the signal sequence of *Herpes simplex* virus glycoprotein D gene (HSV gD) at the N-terminus and transmembrane domain and cytoplasmic region of HSV gD at the C-terminus as previously described^17,18^. The plasmid construct, pHVDR22, used to express PvDBPII on surface of COS7 cells, was described earlier^17^.

COS7 cells were transfected with plasmid constructs designed to express PvRBP1a_30_ and PvDBPII and used for binding assays with reticulocytes and erythrocytes as described earlier^18^. Briefly, 100 μl of 1% erythrocyte or reticulocyte suspensions were added to monolayers of transfected COS7 cells in 900 μl of complete DMEM. The plates were incubated for 1 h at 37 °C and then washed three times with DMEM to remove unbound erythrocytes or reticulocytes. Transfected COS7 cells with rosettes of adherent erythrocytes or reticulocytes were examined by microscopy for presence of rosettes of adherent erythrocytes or reticulocytes. A rosette was defined as 5 or more adherent erythrocytes or reticulocytes bound to a COS7 cell. The number of rosettes was scored in 20 fields at 40X magnification using an inverted microscope.

### Immunofluorescence Assay (IFA) to detect expression of PvRBP1a_30_– HSV gD and PvDBPII-HSV gD on COS7 cells

Transfected COS7 cells were tested for expression of chimeric PvRBP1a_30_– HSV gD and PvDBPII-HSV gD chimeric proteins by IFA using monoclonal antibody DL6 (mAb DL6) against a proline rich region of HSV gD as described earlier^17^. Briefly, the transfected cells were washed with PBS and fixed in 2% formaldehyde for 15 min at RT. The fixed cells were then incubated with mAb DL6 (1:1000 dilution in PBS containing 0.1% BSA) for 1 h at room temperature. Untransfected COS7 cells were incubated with mAb DL6 as control. The cells were washed three times with PBS and then incubated with FITC-conjugated goat anti-mouse antibodies (Sigma Aldrich Pvt. Ltd., USA) (1:100 dilution) for 1 h. The slides were visualized at magnification of 100X using a fluorescence microscope (Nikon Eclipse TE 2000U).

### Production of recombinant PvRBP1a_30_ and raising antibodies against PvRBP1a_30_ in mice and rabbits

A synthetic gene encoding the amino acid sequence of PvRBP1a_30_ (352 aa–599 aa) from *P. vivax* SalI strain was codon optimized for recombinant protein expression in *E. coli*^19^. Restriction sites *Nco*I & *Xho*I were introduced at the 5′ and 3′ ends of the synthetic gene respectively, to enable cloning in the *E. coli* expression vector pET28a (+) (Novagen, USA). *E. coli* Shuffle 30 cells (New England Biolabs, USA) were transformed with expression plasmid PvRBP1a_30_ pET28a (+) and cultured at 30 °C to mid-log phase with OD_600_ of 0.6–0.8. Expression of recombinant PvRBP1a_30_ was induced with 1 mM IPTG at 16 °C overnight. Following induction, the cells were harvested by centrifugation at 4000 g and lysed by sonication. Soluble cytosolic fraction was separated by centrifugation. Recombinant PvRBP1a_30_ was purified from soluble cytosolic fraction under native conditions by metal affinity chromatography (Ni-NTA) followed by gel permeation chromatography using a Superdex 75 column (GE Healthcare, Sweden).

Recombinant PvRBP1a_30_ was used to raise antisera in mice and rabbits. Balb/C mice and New Zealand White rabbits were immunized intramuscularly with 25 μg and 100 μg respectively of the recombinant protein formulated with complete Freund’s adjuvant (Sigma, USA) for priming on day 0 followed by two booster immunizations with recombinant proteins formulated with incomplete Freund’s adjuvant on days 28 and 56. Sera were collected on days 0, 14, 54 and 74 and used to determine ELISA end point titers for recognition of recombinant PvRBP1a_30_. End point titers were calculated as the last dilution that gives OD_492_ values by ELISA above background values for day 0 sera (i.e. mean OD_492_ + 2 SD for day 0 sera, where SD is standard deviation). Total IgG was purified from rabbit sera using Protein G affinity column (GE Healthcare, Sweden), dialyzed with RPMI 1640 medium and tested in binding and invasion inhibition assays as described below.

### Reticulocyte/erythrocyte binding assay with recombinant PvRBP1a_30_ and PvDBPII using flow cytometry

Recombinant PvRBP1a_30_ was produced as described above. Recombinant PvDBPII was produced as described previously^20^. 1 × 10^7^ human erythrocytes or enriched reticulocytes were incubated with 4 µg of recombinant PvRBP1a_30_ or PvDBPII for 2 h at 25 °C. Following incubation, the samples were washed with PBS, incubated with rabbit antisera against the respective recombinant proteins (diluted 1: 500 in PBS) for 1 h at room temperature. The samples were washed two times with PBS, incubated with fluorescein isothiocyanate (FITC) conjugated anti-rabbit IgG (Sigma, USA) goat antibodies for 1 h at room temperature, washed two times with PBS and used for analysis by flow cytometry using a Becton Dickinson FACS Calibur. For binding inhibition assays, the recombinant proteins were incubated with different concentrations of purified IgG (10 µg/ml to 100 µg/ml) followed by incubation with erythrocytes or reticulocytes. For each experiment, data was acquired for 50,000 cells with the same FACS settings for each condition. Data was analyzed using Flow Jo software. The desired populations were gated based on size and granularity.

### Detection of native PvRBP1a in late schizonts and merozoites by IFA

Blood from patients infected with *P. vivax* was layered on a 70% Percoll gradient and centrifuged at 1,000 g for 10 mins to enrich viable schizont-infected erythrocytes. Thin smears of late stage *P. vivax* schizonts were made on glass slides, air dried and fixed in methanol for 20 mins at −20 °C. Following blocking with 3% BSA, smears were incubated with anti-PvRBP1a_30_ mouse sera (1:50 dilution) and co-incubated with anti-PvDBPII rabbit sera (1:50 dilution) as micronemal marker for 1 h, followed by 3X washes with PBS. After washing, smears were probed with a mixture of Alex Flour 488 conjugated goat anti-mouse IgG (H + L) secondary antibodies (1:500) and Alex Flour 594 conjugated goat anti-rabbit IgG (H + L) secondary antibodies (1:500) respectively (Molecular Probes, USA) for 1 hr followed by three washings with PBS. Slides were air-dried, stained with DAPI (Molecular Probes, USA) and treated with anti-Fade (Molecular Probes, USA) to reduce loss of signal. Slides were visualized on a Nikon A1R Confocal microscope (Nikon, Japan) using NIS-elements software.

### Reticulocyte invasion assays using *P. vivax* field isolates

*P. vivax* field isolates were collected in Ratanakiri (Northeast Cambodia) between November 2015 and February 2016 from *P. vivax* mono-infected patients seeking treatment following approval of protocol by relevant ethics committees and after obtaining written informed consent from volunteers. Blood was collected in lithium-heparin tubes and immediately processed on site. Blood was centrifuged, plasma was removed, cells were washed once with RPMI1640 and leukocytes were depleted using NWF filters (Antoshin). Two volumes of Glycerolyte 57 (Baxter, USA), was added drop by drop to one volume of packed RBC pellet with constant agitation prior to aliquoting in 2 ml cryovials and immediate freezing in liquid nitrogen.

Two to three cryovials per isolate were thawed using step-wise addition of NaCl solution. Parasites were then incubated at a final hematocrit of 5% under 5%CO_2_ and 5%O_2_ atmosphere in complete RPMI1640 medium (RPMI1640 (Gibco, USA) supplemented with 0.5% Albumax II (Gibco, USA), 2.5% human serum, 25 mM HEPES (Gibco, USA), 20 μg/ml gentamycin (Sigma, USA) and 0.2 mM hypoxanthine (C-C Pro)). Once parasites reached mature schizont stage, cells were re-suspended in PBS, 2 mM EDTA, 0.5% BSA (PEB) and passed on a CS column (Miltenyi Biotech, France) set on a VarioMACS (Miltenyi Biotech, France). Schizonts were eluted with PEB, washed three times in complete RPMI and immediately used for *in vitro* invasion assay.

Placental-side cord blood was collected in lithium-heparin tubes (20 ml) and processed within 6–8 h post-collection. Blood was centrifuged, plasma was removed, cells were washed once with RPMI1640 and leukocytes were depleted using cellulose filtration (Sigmacell cellulose, Sigma, USA). Reticulocytes were enriched on a 70% isotonic Percoll cushion (Sigma, USA), washed three times with RPMI1640 and cryopreserved as described above. On the day of the invasion assay, reticulocytes were thawed as described above and labeled with 5-(and 6)-carboxyfluoresceindiacetate – succinimidylester (CFDA-SE) according to the manufacturer’s instructions (Life Technologies, USA). Cells were then washed three times in RPMI1640 and re-suspended in complete RPMI for immediate use.

Reticulocyte invasion assays were performed in 100 μl final volume in 96-well plates. Schizonts and CFDA-SE-labeled reticulocytes were mixed at a ~1:6 ratio and incubated in RPMI 1640 medium at a final hematocrit of ~1% under 5%CO_2_ and 5%O_2_ atmosphere in presence of IgGs. A no-inhibitor control with only RPMI 1640 without any IgGs was included in every assay. Single IgGs were tested at 5 mg/ml, the combination of anti-PvDBPII and anti-PvRBP1a included both antibodies with each at concentration of 5 mg/ml. IgG purified from pre-immune control seraIgGs (PIS) were tested at 10 mg/ml concentration. Invasion was assessed ~8 h after assay setup. Cells were harvested, incubated with Hoechst 33342 DNA dye (Sigma) for 15 min and analyzed by flow cytometry on a Cube8 FACS instrument (Partec). Invasion was scored as the percent of Hoechst positive cells (FL4 parameter) among CFDA-SE positive cells (FL1 parameter). Invasion inhibition for each IgG was expressed relative to the no-inhibitor controls. The percent inhibition was calculated using the formula below. Each condition was tested in 4 independent experiments.

$${\rm{Invasion}}\, \% =\frac{100\times {\rm{Number}}\,{\rm{of}}\,{\rm{events}}\,{\rm{positive}}\,{\rm{for}}\,{\rm{both}}\,{\rm{Hoechst}}\,{\rm{and}}\,{\rm{CFDA}} \mbox{-} {\rm{SE}}}{{\rm{Number}}\,{\rm{of}}\,{\rm{events}}\,{\rm{positive}}\,{\rm{for}}\,{\rm{CFDA}} \mbox{-} {\rm{SE}}}$$

$${\rm{Inhibition}}\, \% =100 \mbox{-} {\rm{Invasion}} \% $$

### Ethics Statement

Collection of blood from *P. vivax* infected patients and collection of reticulocytes from cord blood to perform *in vitro* invasion assays was approved by the National Ethics Committee for Health Research of the Cambodian Ministry of Health (Approval 0364NECHR delivered on the 29^th^ December 2014). An informed written consent was obtained from all patients or their parents/guardians if aged less than 18 years old prior to enrollment. All procedures were carried out in strict accordance with relevant guidelines and regulations.

The study was also carried out in strict accordance with the recommendations in the Guide for the Care and Use of Laboratory Animals of the Institut Pasteur (http://webcampus.pasteur.fr/jcms/c_283578/procedures-approuvees-par-le-comite-d-ethique) and complied with the European Union guidelines for the handling of laboratory animals (http://ec.europa.eu/environment/chemicals/lab_animals/home_en.htm). The procedures used were approved by the Institut Pasteur animal care and use committee. Animal care and handling was approved by the Ministère de l′Enseignement Supérieur de la Recherche et de l′Innovation (Ref. APAFIS 8845-2017122117082418v3). All animal experiments were planned and executed in order to minimize animal suffering.

### Data availability

The authors confirm that materials, data and protocols will be made available to readers upon request.

## Results

### Mapping the receptor-binding domain of PvRBP1a protein

**Figure 1:**
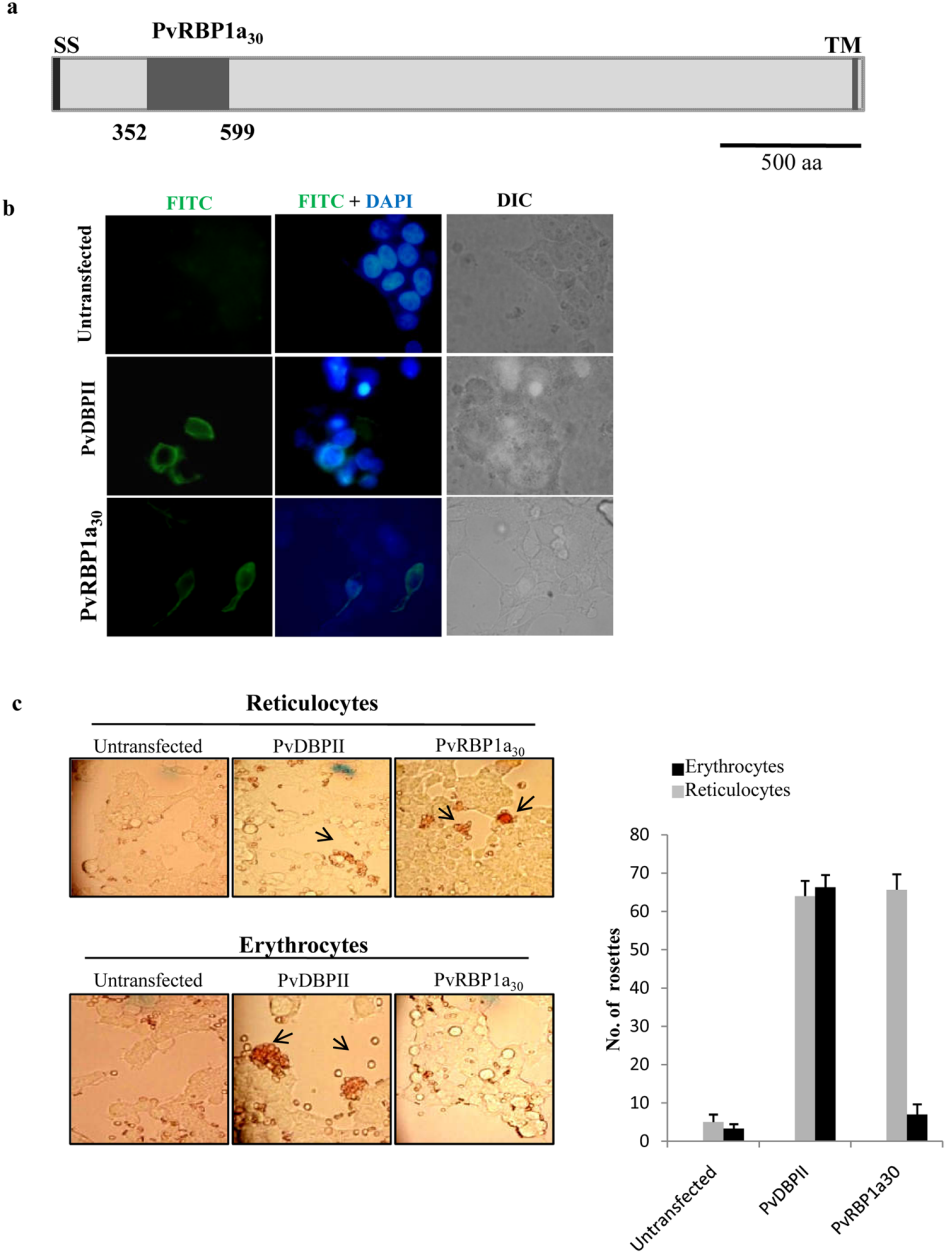
Binding assays with PvRBP1a_30_ and PvDBPII expressed on surface of COS7 cells. (**a**) Schematic representation of PvRBP1a. Signal sequence (SS), PvRBP1a_30,_ predicted 30 kD reticulocyte binding domain of PvRBP1a (352 aa to 599 aa), transmembrane domain (TM). (**b**) Immunofluorescence assays showing expression of PvRBP1a_30_ (green) and PvDBPII (green) on the surface of transfected COS7 cells. PvDBPII was included as a positive control. DNA staining dye DAPI (4′,6-diamidino-2-phenylindole) (blue) was used to stain all COS7 cells. DIC, differential interference contrast, FITC, fluorescein isothiocyanate (green). (**c**) Rosetting assays using reticulocyte enriched blood and erythrocytes with transfected COS7 cells expressing PvRBP1a_30_ or PvDBPII on their surface. Number of rosettes of erythrocytes or reticulocytes was scored in 20 fields at 40X magnification (mean ± standard deviation from 3 independent experiments). PvRBP1a_30_ binds reticulocytes but not erythrocytes whereas PvDBPII binds both erythrocytes and reticulocytes.

DNA encoding a ~30 kD region of PvRBP1a (Fig. 1a) that shares homology with the receptor-binding domain of the homologous *P. falciparum* invasion protein PfRH4 (Figure S2) was cloned in the mammalian cell expression vector, pRE4, to create a fusion with the signal sequence of HSV gD at the N-terminus and with the transmembrane region and cytoplasmic domain of HSV gDat the C-terminus as described earlier^17,18^. Mammalian COS7 cells were transfected with the expression construct and surface expression of recombinant PvRBP1a_30_ was monitored by IFA using mAbDL6^17,18^ against the proline rich region at the C-terminus of the extracellular domain of HSV gD in the fusion protein. Transfected COS7 cells expressed PvRBP1a_30_-HSV gDfusion protein on the cell surfaceas confirmed by IFA (Fig. 1b). The mAb DL6 did not react with untransfected COS7 cells. COS7 cells transfected with PvDBPIIalso showed expression of the recombinant protein on COS7 cellsurface consistent with previous studies^17^. Transfected COS7 cells were tested for their ability to bind erythrocytes and reticulocytes. The number of COS7 cells with rosettes of adherent reticulocytes or erythrocytes for each construct was determined in 20 fields at a magnification of 40X (Fig. 1c). PvRBP1a_30_ showed significantly higher binding with enriched reticulocytes compared to erythrocytes. In contrast,PvDBPII formed rosettes with both erythrocytes as well as reticulocytes as its receptor, DARC, is expressed on both cell types. No rosettes were seen withuntransfected cells ruling out the possibility of non-specific binding.

### Expression, purification and characterization of recombinant PvRBP1a_30_

**Figure 2:**
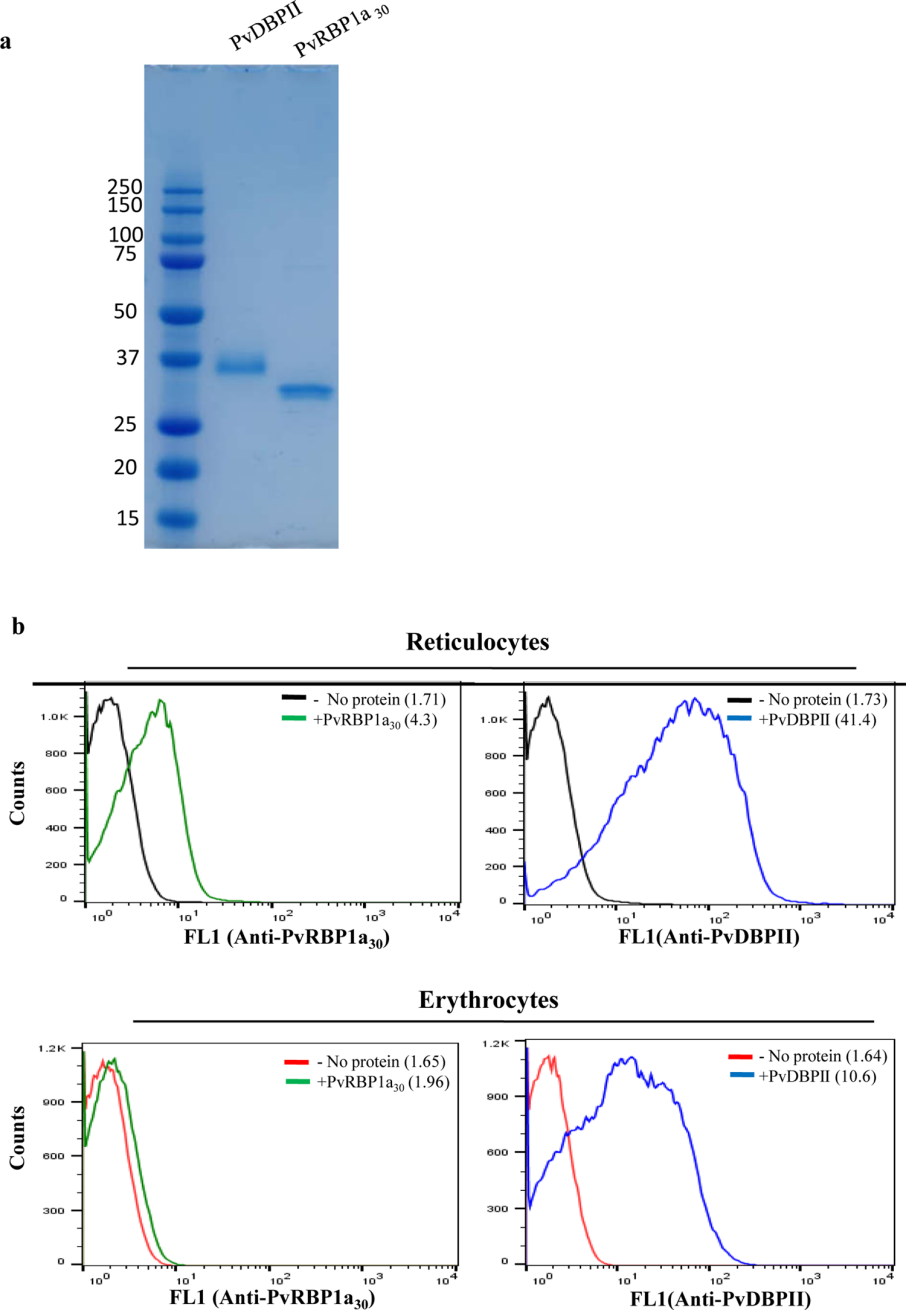
Functional characterization of recombinant PvRBP1a_30_ and PvDBPII. (**a**) Purified recombinant PvRBP1a_30_ and PvDBPII (5 μg each) were separated by SDS-PAGE and detected by Coomasie staining. (**b**) Binding of recombinant PvRBP1a_30_ and PvDBPII to reticulocytes and mature erythrocytes was detected by flow cytometry using specific sera raised against PvRBP1a_30_ and PvDBPII. PvDBPII bound both erythrocytes as well as reticulocytes. PvRBP1a_30_ only bound reticluocytes. Mean fluorescence intensities (MFI) for each graph are shown in parenthesis.

A synthetic gene encoding the binding domain of PvRBP1a (aa 352 to aa 599) with a C-terminal 6X His tagwas codon optimized and used for expression of recombinant PvRBP1a_30_ in *E. coli*. Recombinant PvRBP1a_30_, whichwas expressed as a soluble protein in *E. coli*, was purified by metal affinity chromatography and further purified to homogeneity by gel permeation chromatography (Fig. 2). The functional activity of recombinant PvRBP1a_30_ was tested in binding assays with reticulocytes and erythrocytes using flow cytometry to detect binding (Fig. 2). Recombinant PvDBPII produced as described earlier^20^ was used as a positive control for the binding assays (Fig. 2). Recombinant PvRBP1a_30_ bound reticulocytes but not mature erythrocytes (Fig. 2). In contrast, recombinant PvDBPII bound both reticulocytes and erythrocytes (Fig. 2).

### PvRBP1a localizes to the apical end of merozoites

**Figure 3:**
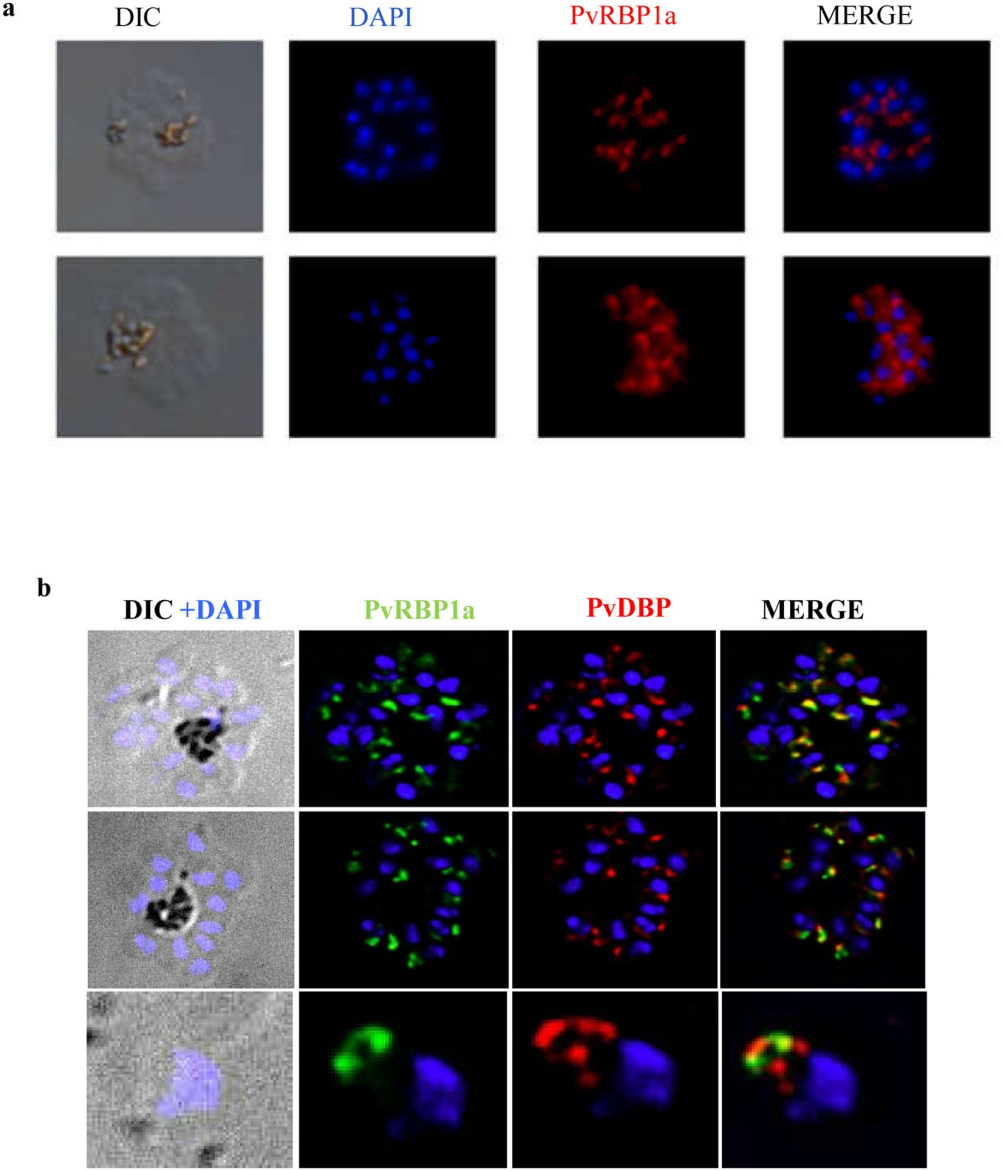
Antibodies against PvRBP1a30 and PvDBPII recognize native protein in *P. vivax* schizonts and merozoites. (**a**) Anti-PvRBP1a_30_ rabbit sera recognize native PvRBP1a in *P. vivax* schizonts. (**b**) Mouse sera raised against PvRBP1a_30_ and rabbit sera raised against PvDBPII were used for immunofluorescence assays with *P. vivax* late stage schizonts and free merozoites. PvRBP1a and PvDBP both localize at the apical end of merozoites.

Recombinant PvRBP1a_30_ was used to raise antisera in rabbits. Rabbit sera against PvRBP1a_30_ was tested for recognition of recombinant PvRBP1a_30_ by ELISA. Rabbit sera collected at day 70 after one priming and two booster immunizations with recombinant PvRBP1a_30_ had high ELISA recognition endpoint titer of 1: 160,000 (Figure S3). Anti-PvRBP1a_30_ rabbit sera also recognized native PvRBP1a in *P. vivax* schizonts by IFA (Fig. 3). PvRBP1a is localized at the apical end of merozoites in mature *P. vivax* schizonts. Mouse sera raised against PvRBP1a_30_ and rabbit sera raised against PvDBPII were used in co-localization experiments to define the location of PvRBP1a in merozoites (Fig. 3). PvDBP and PvRBP1a appear to co-localize at the apical end of merozoites suggesting that PvRBP1a may be located in the micronemes (Fig. 3).

### Antibodies against receptor-binding domains of PvRBP1a and PvDBPII inhibit reticulocyte binding by recombinant proteins

**Figure 4:**
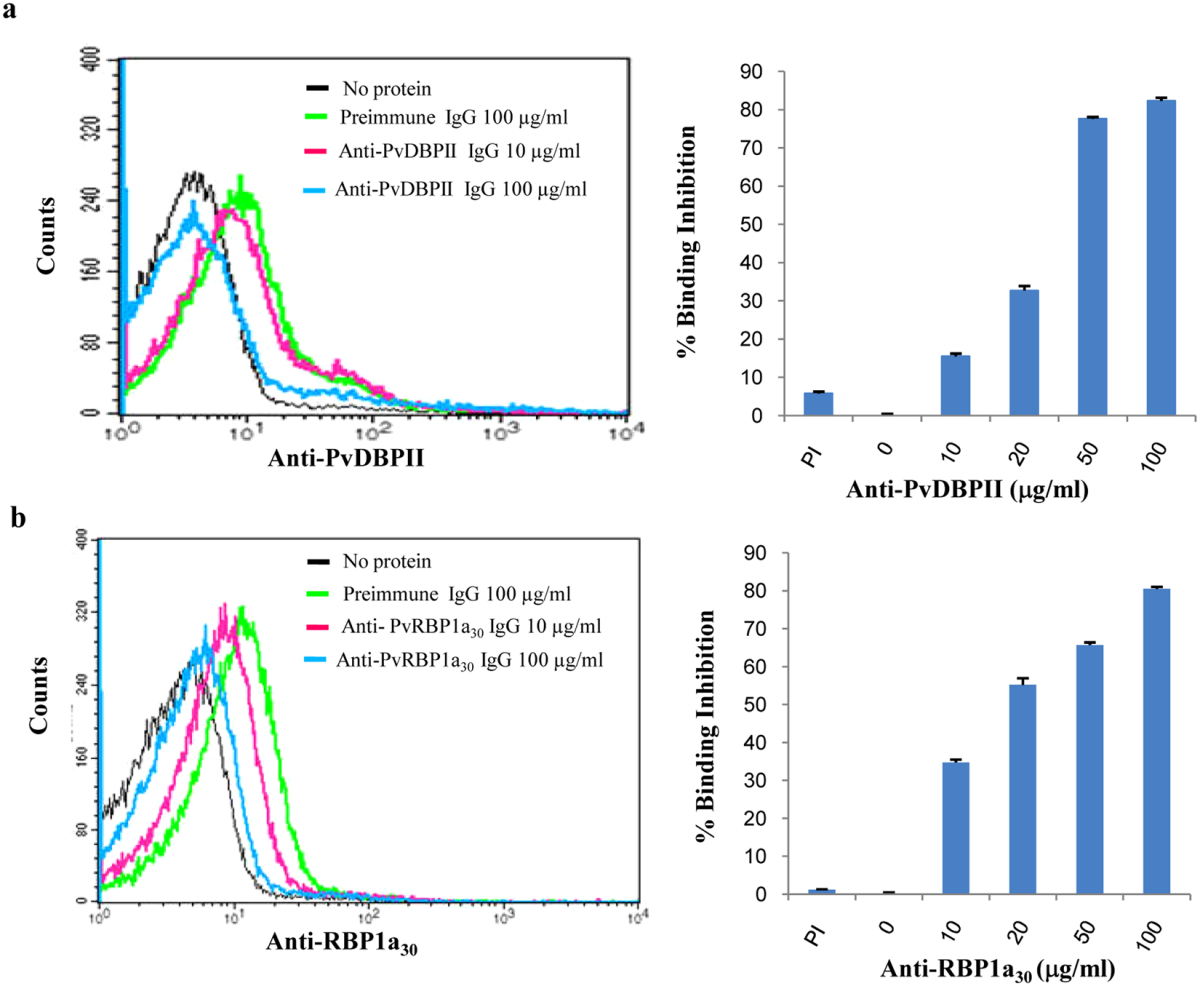
Rabbit antibodies against PvRBP1a_30_ and PvDBPII block reticulocyte binding. (**a** and **b**). Inhibition of PvDBPII and PvRBP1a_30_ binding to reticulocytes by specific antibodies. IgGs purified from rabbit sera raised against recombinant PvDBPII (**a**) or PvRBP1a_30_ (**b**) were tested for inhibition of binding to reticulocytes at different concentrations. IgGs purified from pre-immune rabbit sera (PI) were used as control.

To determine whether antisera against PvRBP1a_30_ or PvDBPII can block the reticulocyte binding activity of PvRBP1a and PvDBPII respectively, we performed binding assays with recombinant PvRBP1a_30_ and PvDBPII using reticulocytes in the presence of different concentrations of total IgG purified from rabbit sera. IgG purified from anti-PvRBP1a_30_ rabbit sera blocked binding of recombinant PvRBP1a_30_ protein with reticulocytes in a dose dependent manner with ~ 90% inhibition at total IgG concentration of 100 μg/ml (Fig. 4). These results showed that purified antibodies specifically recognize PvRBP1a_30_ and inhibit its interaction with reticulocytes. We also observed dose dependent inhibition of binding of PvDBPII to RBCs with IgG purified from anti-PvDBPII rabbit sera (Fig. 4).

### Invasion inhibitory activity of antibodies raised against receptor-binding domains of PvRBP1a_30_ and PvDBPII

**Figure 5:**
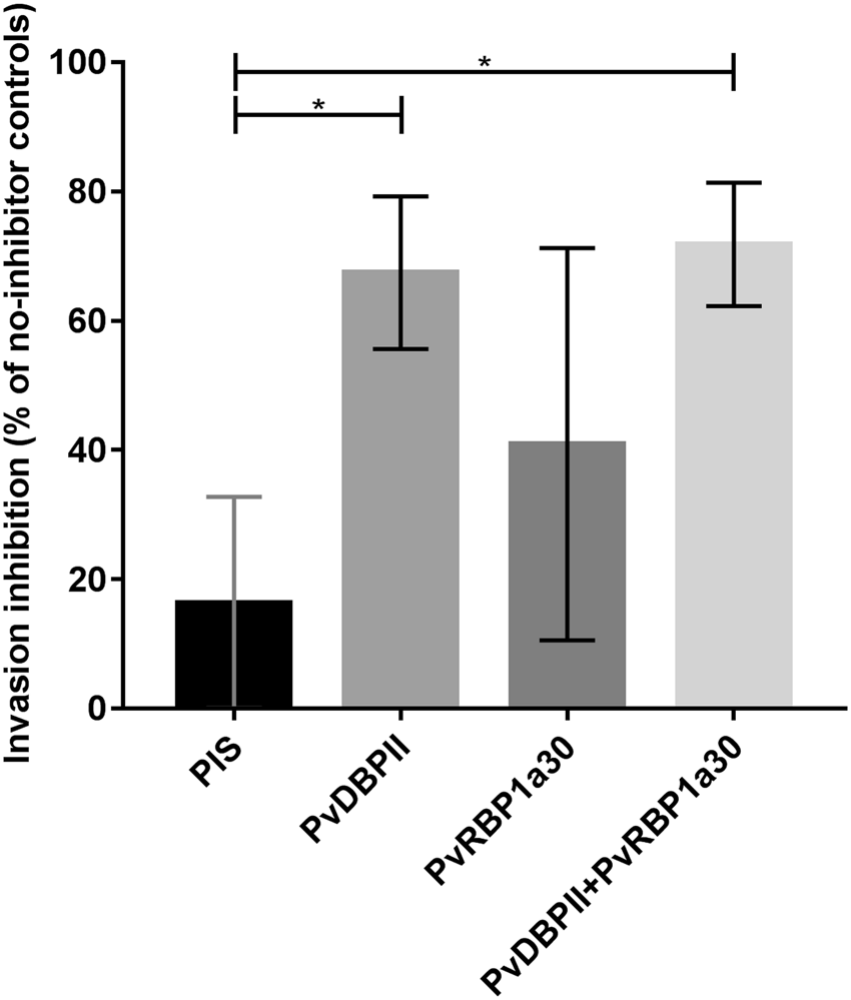
Inhibition of *P. vivax* invasion into reticulocytes with rabbit IgGs against PvRBP1a_30_ and PvDBPII. Antibodies against PvDBPII and PvRBP1a_30_ were purified from rabbit sera and incubated with reticulocytes in presence of *P. vivax* schizonts. Newly invaded rings were scored by flow cytometry using DNA intercalating dye H33342. IgG against PvDBPII inhibits reticulocyte invasion whereas IgG against PvRBP1a has no effect. Combining IgGs against PvRBP1a_30_ and PvDBPII did not improve inhibition of invasion achieved by anti-PvDBPII IgG alone. (Mann-Whitney U test, *indicates P < 0.05, N = 4 independent biological replicates, bars represent mean ± SD).

Further, we tested if IgG purified from anti-PvRBP1a_30_ and anti-PvDBPII rabbit sera can inhibit reticulocyte invasion by *P. vivax* isolates *in vitro*. *P. vivax* isolates collected from vivax malaria patients were cultured *in vitro* to late schizont stages and incubated with reticulocyte enriched blood in presence of different concentrations of IgG purified from anti-PvRBP1a_30_ and anti-PvDBPII rabbit sera. Anti-PvRBP1a_30_ rabbit IgG and anti-PvDBPII rabbit IgG were tested individually and in combination for inhibition of reticulocyte invasion by *P. vivax*. Total IgG purified from naïve rabbit sera before immunization (pre-immune sera) were used as controls in the invasion assays. While anti-PvDBPII IgG consistently blocked reticulocyte invasion by *P. vivax in vitro*, anti-PvRBP1a_30_ IgG had no statistically significant invasion inhibitory activity (P = 0.343, Fig. 5). Combining IgG against PvDBPII and PvRBP1a_30_ did not provide any additional invasion inhibitory activity over that observed with anti-PvDBPII IgG alone.

## Discussion

The invasion of reticulocytes by *P. vivax* merozoites involves several receptor ligand interactions^6^. *P. vivax* reticulocyte binding proteins belonging to PvRBP1 and PvRBP2 sub-families have been shown to bind reticulocytes and are predicted to be involved in selection of reticulocytes for invasion by *P. vivax* merozoites^14–16^. These proteins were first identified in erythrocyte and reticulocyte binding assays with *P. vivax* short term culture supernatants^14^. The PvRBPs are homologous to *P. falciparum* PfRH proteins that are responsible for determining different invasion phenotypes in *P. falciparum*. Transferrin receptor-1 (Tfr1), an essential housekeeping gene responsible for transport of iron into cells that is primarily found on reticulocytes and is lost as they mature into erythrocytes, is used as a key receptor by *P. vivax* for invasion^21^. PvRBP2b has been shown to bind Tfr1 to mediate invasion^21^. The crystal structure of PvRBP2b is similar to the structure of PfRH5, which binds basigin on RBCs and plays a key role in invasion by *P. falciparum* merozoites^21^. In addition to the PvRBP family, the other important interaction for invasion involves the binding of PvDBP with DARC^17^. The functional erythrocyte binding domain of PvDBP has been shown to lie in the conserved N-terminal cysteine rich region referred to as region II (PvDBPII)^17^.

In the present study, we expressed the 30 kD reticulocyte-binding domain of PvRBP1a (PvRBP1a_30_) on the surface of COS7 cells and tested binding to mature erythrocytes and reticulocytes in rosetting assays. PvRBP1a_30_ shares homology with the erythrocyte binding region of PfRH4 and has been previously shown to have reticulocyte binding activity^22^. PvRBP1a_30_ expressed on the surface of COS7 cells showed specific binding to reticulocytes in rosetting assays (Fig. 1). Reticulocytes were enriched from normal human blood using magnetic beads coupled with anti-CD71 antibodies. This technique exploits the fact that the transferrin receptor, CD71, is expressed on reticulocytes and not on mature erythrocytes.

In order to further confirm the binding of PvRBP1a_30_ to reticulocytes, we expressed this region in *E. coli* as a soluble 30 kD protein with a 6X–His tag at the C-terminus. Purified, recombinant PvRBP1a_30_ was used to immunize mice and rabbits to generate specific antibodies. Immunofluorescence assays show that anti-PvRBP1a_30_ sera specifically recognize native PvRBP1a at the apical end of merozoites in mature schizonts. PvRBP1a appears to co-localize with PvDBP suggesting that it may be located in the micronemes in *P. vivax* merozoites. The reticulocyte-binding activity of PvRBP1a_30_ was tested in binding assays using flow cytometry. PvRBP1a_30_ showed preferential binding to reticulocytes compared to mature erythrocytes. In contrast, PvDBPII binds both reticulocytes and RBCs. The mean fluorescence intensity (MFI) for binding to reticulocytes is higher than to mature erythrocytes (Fig. 2b) due to the higher copy number of DARC expressed on reticulocytes. Next, we evaluated the binding inhibitory potential of antibodies against PvRBP1a_30_ at different concentrations using a flow cytometry based binding assay. Antibodies generated against recombinant PvRBP1a_30_ inhibit binding of PvRBP1a_30_ to reticulocytes in a dose dependent manner. Similarly, antibodies raised against PvDBPII block PvDBPII-binding to both reticulocytes and RBCs. Previous studies have shown that individuals residing in malaria endemic regions develop antibodies to PvRBP1a_30_ and PvDBPII following natural exposure to *P. vivax* infection. These naturally acquired antibodies have been shown to block receptor-binding by PvRBP1a_30_^23^ and PvDBPII^24^. Based on these observations both PvRBP1a_30_ and PvDBPII are considered promising vaccine candidates for *P. vivax*. However, due to the lack of methods for *in vitro* culture of *P*. *vivax* blood stage parasites, antibodies against PvRBP1a_30_ have not so far been tested for inhibition of reticulocyte invasion by *P*. *vivax*. Here, we have used a method for short term *in vitro* culture of *P. vivax* field isolates to test the ability of total IgG purified from rabbit sera against PvRBP1a_30_ and PvDBPII to inhibit *P. vivax* invasion into reticulocytes. Interestingly, while antibodies against PvDBPII inhibit reticulocyte invasion, antibodies against PvRBP1a_30_ do not have a significant impact on reticulocyte invasion by *P. vivax* (Fig. 5). Combining antibodies against PvRBP1a_30_ and PvDBPII does not enhance invasion inhibitory activity. The reasons for lack of invasion inhibitory activity in case of antibodies against PvRBP1a_30_ are not known. It is possible that antibodies to PvRBP1a_30_ are ineffective at blocking invasion due to the presence of alternate pathways in *P. vivax* field isolates. For example, homologs of PvRBP1a such as PvRBP2b, which have been shown to bind CD71 on reticulocytes^21^ may mediate invasion by such alternative pathways. Alternatively, antibodies to PvRBP1a may not have access to the parasite antigen during the process of invasion rendering them ineffective. The observations here on the lack of invasion inhibitory activity of antibodies against PvRBP1a_30_ suggest that it may not be useful to include PvRBP1a_30_ in a multi-component blood stage vaccine for *P. vivax* malaria. In contrast, the observation that antibodies to PvDBPII can inhibit reticulocyte invasion by diverse *P. vivax* isolates validates inclusion of PvDBPII in a vaccine for *P. vivax* malaria.

## Electronic supplementary material

Supplementary Figures S1, S2, S3
