# Supplementary material for: Targeting a Reticulocyte Binding Protein and Duffy Binding Protein to Inhibit Reticulocyte Invasion by Plasmodium vivax
Source: Sci Rep. 2018 Jul 12;8:10511. doi: 10.1038/s41598-018-28757-4 (PMC6043553; doi:10.1038/s41598-018-28757-4)
Supplement: Supplementary file 1 — Supplementary Figures S1, S2, S3 [file 41598_2018_28757_MOESM1_ESM.docx]

**SUPPLEMENTARY DATA**

**Targeting a Reticulocyte Binding Protein and Duffy Binding Protein to Inhibit Reticulocyte Invasion by *Plasmodium vivax***

Sonal Gupta^1,2^, Shailja Singh^2,3,4^, Jean Popovici^5^, Camille Roesch^5^,

Ahmed Rushdi Shakri^1^, Micheline Guillotte-Blisnick^4^, Christèle Huon^4^,

Didier Menard^4,5^ and Chetan E. Chitnis^1,4,*^

^1^International Centre for Genetic Engineering and Biotechnology (ICGEB), New Delhi, India,^2^Special Centre for Molecular Medicine, Jawaharlal Nehru University, New Delhi, India*,*^3^Shiv Nadar University, Gautam Buddha Nagar, India, ^4^Institut Pasteur, Paris, France, ^5^Institut Pasteur of Cambodia, Phnom Penh, Cambodia

**Supplementary Figure Legends**

**Supplementary Figure S1:** Reticulocyte enrichment from normal human blood by immunomagnetic separation. **(A)** Reticulocytes were stained with new methylene blue. The percentage of reticulocytes in normal human adult blood comprises ~1% of total blood cells. After enrichment by magnetic separation the percentage of reticulocytes increased to 50-60%. Reticulocytes can be differentiated from mature erythrocytes by presence of blue black intracytoplasmic granules. **(B)** Analysis of blood before (blue) and after (red) reticulocyte enrichment by flow cytometry. Reticulocyte enriched blood stained with mouse anti-CD71 IgG conjugated to phycoerythrhin (anti-CD71 PE) showed ~51% CD71 positive reticulocytes (R2) after enrichment.

**Supplementary Figure S2:** Mapping of a 30kDa reticulocyte binding domain of PvRBP1 (PvRBP1a_30_), chosen on the basis of homology with erythrocyte binding domain of PfRH4. ClustalW alignment of PfRH4 (GenBank accession no. AF432854.1) *dark grey* and PvRBP1 (GenBank accession no. Q00798) *light grey*. *, identical residues; :, conserved substitutions; ., semiconserved substitutions.

**Supplementary Figure S3:** Immunogenicity of recombinant PvRBP1a_30_ protein. **(A)** End point titer of rabbit serum raised against PvRBP1a_30_ protein was determined by standardized ELISA. Sera were collected at Day 14, Day 42 and Day 74. ELISA reactivity (mean + SD) of all sera are shown at different dilutions. End point titer of Day 74 sera was observed to be 1:160,000. **(B)** Day 74 anti-PvRBP1a_30_ rabbit sera reacts with 30kDa protein band of recombinant PvRBP1a_30_ by Western blotting.

**Supplementary Figure S1**.

**a**

**Before Enrichment After Enrichment**


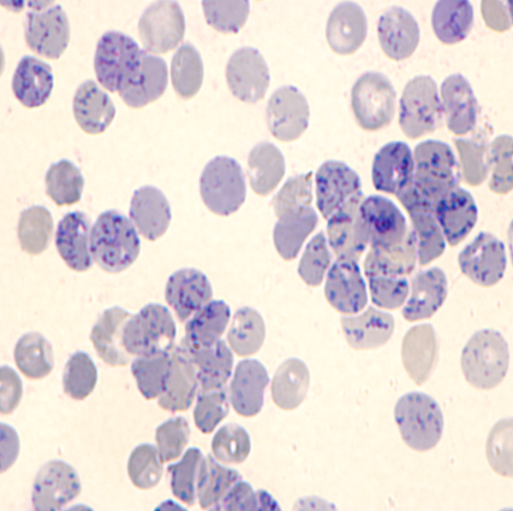


~ 60% reticulocytes


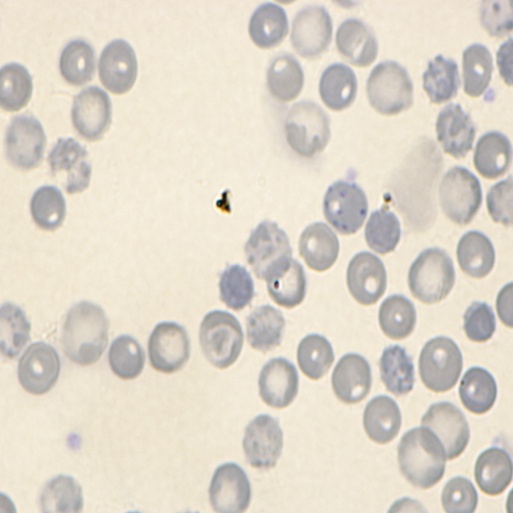


~ 1% reticulocytes

**b**


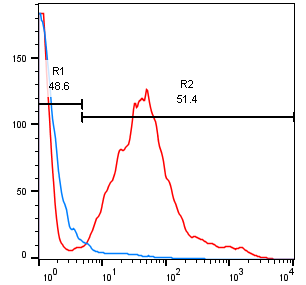

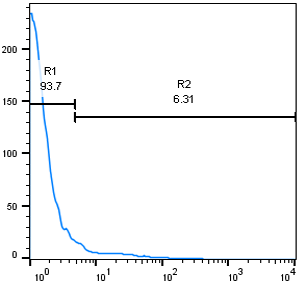


**FL2 (Anti CD71 PE)**

**Count**

**After enrichment**

**Before enrichmentCounted with anti CD71**

**Supplementary Figure S2.**


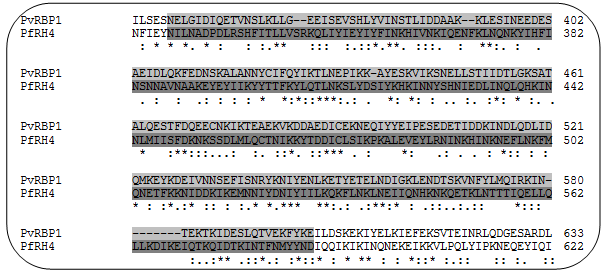


**Supplementary Figure S3.**

**a**

**Optical Density at 492 nM (OD**

**Sera Dilutions**

**b**

**Anti-RBP1a_30_ Day 74**

**Prebleed**

**30 kDa**

**72**


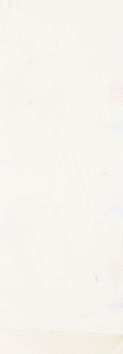

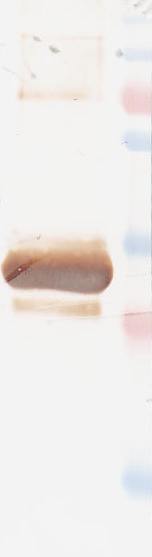


**55**

**34**

**25**
